# Supplementary material for: Complexin-1 regulated assembly of single neuronal SNARE complex revealed by single-molecule optical tweezers
Source: Commun Biol. 2023 Feb 7;6:155. doi: 10.1038/s42003-023-04506-w (PMC9905088; doi:10.1038/s42003-023-04506-w)
Supplement: Supplementary file 2 — Supplementary Information [file 42003_2023_4506_MOESM2_ESM.pdf]

Supplementary Information for

Complexin-1 regulated assembly of single neuronal SNARE complex by single-molecule optical tweezers

*Tongrui Hao[*1, 2], Nan Feng[1,2], Fan Gong[3], Yang Yu[3], Jiaquan Liu[*1], Yu-Xuan Ren[*4]*

1 State Key Laboratory of Molecular Biology, Shanghai Institute of Biochemistry and Cell Biology, Center for Excellence in Molecular Cell Science, Chinese Academy of Sciences, Shanghai 200031, China

2 University of Chinese Academy of Sciences, Beijing 100049, China

3 National Facility for Protein Science in Shanghai, Zhangjiang Lab, Shanghai Advanced Research Institute, Chinese Academy of Sciences, Shanghai 201210, China.

4 Institute for Translational Brain Research, Shanghai Medical College, Fudan University, Shanghai 200032, China

***Correspondence should be addressed to**

haotongrui2018@sibcb.ac.cn (T. H.); liujiaquan@sibcb.ac.cn (J. L.); yxren@fudan.edu.cn (Y. R.)

1. Open distances for each state of SNARE in presence of mutations of CpxI

The open distance for each of the CpxI mutants referenced to state 2, e.g., the open distance for state 2 is assumed to be 0. All the open distances are expressed in nanometer, while the numbers in the brackets expressed the standard deviation. The Average force records the average applied force on the SNARE complex when the trap separation keeps fixed. For the* marked molecular, they have no state 2 after the introduction of Cpx, the open distance for state 2 before the addition of Cpx is assumed to be 0 in this condition.

	Average force[pN]	2 (unit: nm)	3 (unit: nm)	4 (unit: nm)	5 (unit: nm)
Without CpxI	20.16(0.78)	0	4.67(2.0)	7.02(2.3)	14.71(2.0)
FL C-terminal	19.50(0.62)	*	--	6.94(2.4)	14.25(2.0)
FL Middle-clamped	19.76(0.31)	*	4.9(1.6)	7.2(1.7)	--
FL Linker-open	20.47(0.72)	0	4.3(2.1)	--	14.1(2.4)
1-83aa	20.67(0.99)	0	5.97(2.2)	--	--
26-83 Linker-open	20.46(0.65)	0	6.2(2.2)	--	15.0(2.2)
26-83 C-terminal	21.15(0.97)	*	--	9.6(2.2)	15.0(1.9)
1-83+83-134 Linker-open	20.25(0.74)	0	4.9(2.3)	--	13.9(2.7)
1-83+83-134 (C-terminal)	18.65(0.67)	0	5.1(1.8)	8.1(1.9)	13.6(2.0)
1-83+83-134 (Middle-clamped)	18.53(0.57)	*	6.5(2.3)	10.0(2.2)	--

Supplementary Table 1. Open distance of the SNARE complex in the absence and presence of the mutants of CpxI.

2. Schematic model of the extension changes of the SNARE complex in its different assembly states

The extension of the protein-DNA handle tether, X, is the sum of the extensions of the DNA handle, x_{DNA}, the unfolded polypeptide portion of SNARE complex, x_p, and the core structure, h, i.e.,

$$X = x_{DNA} + x_p + h, \quad (1)$$

where h is assumed as the spatial length of the folded portion (such as coiled coil) projected along pulling direction, which also contributes to the final extension. The x_{DNA} is stable under the same average force F_i (fixed trap), so we focus on the protein extension, i.e., $x_p + h$, which we named $x_i = x_p + h, i = 1, \ldots, 5$. In the fully folded state (native state), $h = 2$ nm is determined from the x-ray structure of the protein[1], whereas for the fully unfolded state, $h = 0$. Then $l_i,\ i = 1, \ldots, 5$, is the contour length of the unfolded polypeptide in different SNARE assembly states, $\alpha = 0.15/0.365 = 0.41$ is the ratio of the contour length of an amino acid (aa) in the helical conformation to that in the coil conformation. The extension of the unfolded polypeptide is related to the contour length and force through the Marko-Siggia formula. The contour length difference at this force point is evaluated based on the model shown in Fig. S1 as

$$\Delta l_i = \Delta x_i / r. \qquad (2)$$

where Δx_i is the extension difference between the two states now under the same tension F_i .

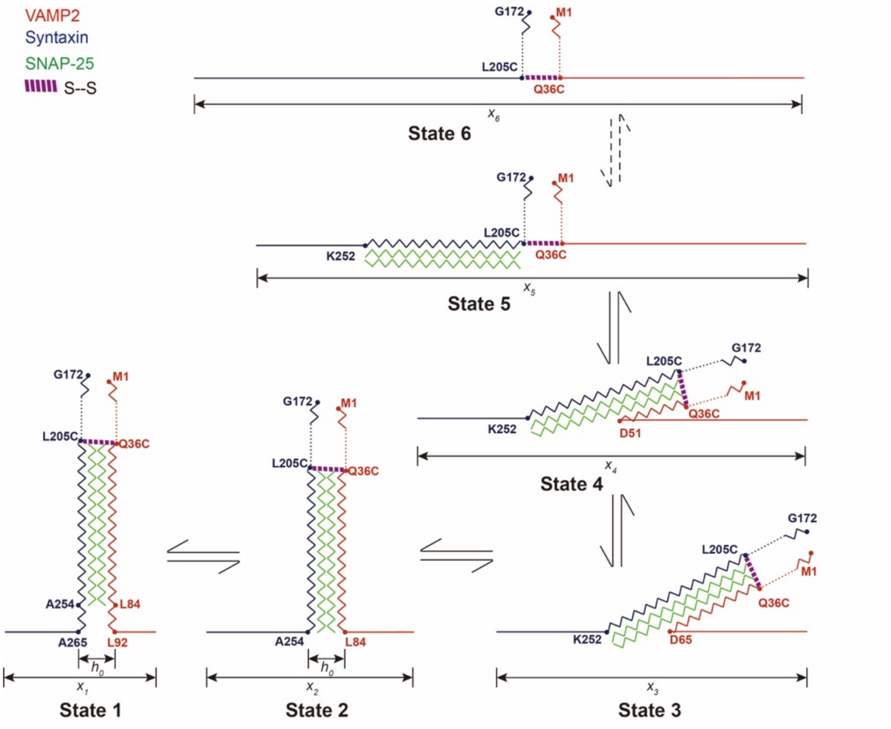

Supplementary Figure 1. Schematic model of the extension changes of the SNARE complex in its different assembly states.

Finally, the average of these contour length differences is calculated. In the case of the α-helix, every 3.6 amino acid residues, the helix rises by a circle, the pitch is 0.54nm, and the span of each residue is 0.15nm. When the polypeptide chain is fully extended, the length of each amino acid residue is 0.365 nm.

Protein extension for state 1:

$$x_1 = l_1 r + h_0 \qquad (3)$$

Protein extension for state 2:

$$x_2 = l_2 r + h_0 \qquad (4)$$

Protein extension for state 5:

$$x_5 = l_5 r + (l_6 - l_5)\alpha \quad (5)$$

Protein extension for state 6:

$$x_6 = l_6 r \qquad (6)$$

We could get the number of unfolded amino acids ($N_i, i = 1{,}6$) is 34 aa and 147 aa and $h_0 = 2nm$ (the diameter of fully zippered four-helix-bundle) from our experiment design and the crystal structure of SNARE complex [1, 2]. Therefore, the contour lengths of the unfolded polypeptides are that $l_1 = 12.41\ nm$, and $l_6 = 53.69\ nm$.

We experimentally measured a change of protein extension length $x_6 - x_1$ of 19.71 nm. Considering the known parameter h_0, from Eques (2) and (5), we could calculate that $r = 0.526$ and $x_1 = 8.53\ nm$.

From the open distance of state 2-6 to state 1 in our experiment, we can further calculate the protein extension for each of those states: $x_2 = 11.53\ nm,\ x_3 = 16.2\ nm, x_4 = 18.55\ nm,\ x_5 = 26.24\ nm,\ x_6 = 28.24\ nm$. The number of unfolded amino acids ($N_i, i = 2{,}5$) in each state is 50, 100 correspondingly, therefore the contour lengths of the unfolded polypeptides are $l_2 = 18.12\ nm$, $l_5 = 36.44\ nm$ respectively.

Specifically, the Syntaxin unfolded 47 aa from state 5 to state 6. As Syntaxin contributed 49 aa in the state 2 (A254 to L205C), 2 aa unfolded between state 2 and state 3.

Since the crystal structure analysis only tackles the ensemble averaged conformations in the absence of mechanical force, the protein extension changes in two intermediate States 3 and 4 can only be inferred through the following,

Protein extension for state 3:

$$x_3 = l_3 r + (l_3 - l_2 - 2 \times 0.365)\alpha \quad (7)$$

Protein extension for state 4:

$$x_4 = l_4 r + (l_4 - l_3)\alpha \quad (8)$$

We can calculate $l_3 = 25.66\, nm$, $l_4 = 31.06\, nm$, which means that the number of unfolded amino acids ($N_i, i = 3,4$) in each state is 71 and 85 respectively. We could get the amino acids sites of state 3, 4 in the Fig. SR1, in which VAMP unfolded 19 aa (L84 to D65) from state 2 to state 3, corresponding to the unzippering of +8 layer to +3 layer in VAMP (+2 layer remain folded, Fig. 1d, state 3). For state 3 to 4, VAMP unfolded 14 aa (D65 to D51), corresponding to the unzippering of +2 layer to -1 layer in VAMP (-2 layer remain folded, Fig. 1d, state 4).

3. Purification and pre-assemble of proteins

The synaptic SNARE complex consists of VAMP2 (1-92, C2A, Q36C), syntaxin 1 (172-265, C173A, L209C), and SNAP25 (1-206). The proteins were then expressed in E. coli BL21(DE3) cells and purified as described in the manual of ChampionTM pET SUMO Expression System (Invitrogen).

Ternary SNARE complexes were formed by mixing syntaxin, SNAP25 and VAMP2 proteins with 3:4:5 molar ratios in 25 mM HEPES, 150 mM NaCl, 2mM TCEP, pH 7.7 and the mixture was incubated at 4 °C for 30 min. Formation of the ternary complex was confirmed by SDS polyacrylamide gel electrophoresis. Excessive SNARE monomers or binary complexes

were removed from the ternary complex by further purification through Ni-NTA resin using the His-Tag on the SNAP25 molecule.

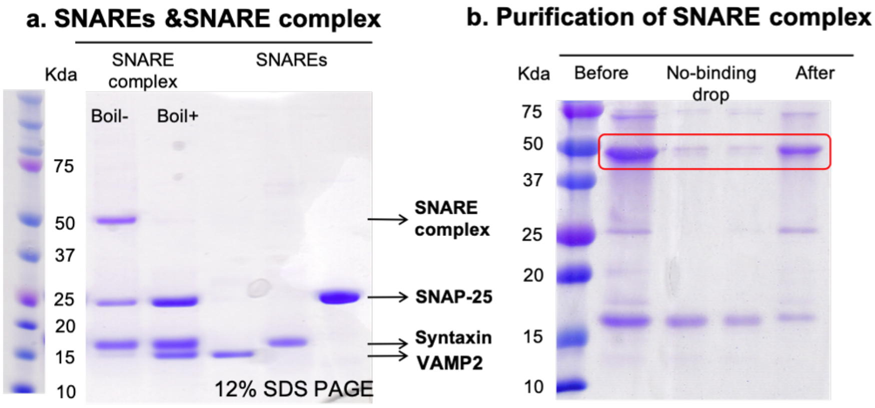

Supplementary Figure 2. Purification and pre-assemble of proteins. **a** Purification and pre-assemble of SNARE Complex, formation of the ternary complex was confirmed by SDS polyacrylamide gel electrophoresis. **b** Purification of SNARE complex by Ni-NTA, SNARE complex was marked by red box.

4. Interaction test between SNAREs and CpxI

The Binding assay between SNAREs and complexin is completed (FigS2a: SNARE monomers, FigS2b: SNARE complex). Among them, the mole ratio of CpxI and SNAREs is 5:1 (due to the larger time cost of SNARE complex sample acquisition, CpxI is selected as excess protein), and they were incubated for 1hr at room temperature. The loading quantity of the same protein in all swimming lanes is strictly quantitative control, and the same stock buffer containing TCEP (strong reducing agent) is added in advance for processing. In Figure S2a, the first lane is CpxI, the second/forth/sixth lane is Syntaxin/VAMP/SNAP25, the third/fifth/seventh lane is SNAREs and CpxI after incubation. For we did not see significant difference on the gel, complexin does not binding to any SNARE monomers. In Figure 2Sb, the first lane is SNARE complex molecule, the second lane is SNARE complex and CpxI after incubation, and the third lane is CpxI protein. The complex-SNARE complex shows a new band on the native page. It is proven that the SNARE complex used in the experiment can stably combine with CpxI.

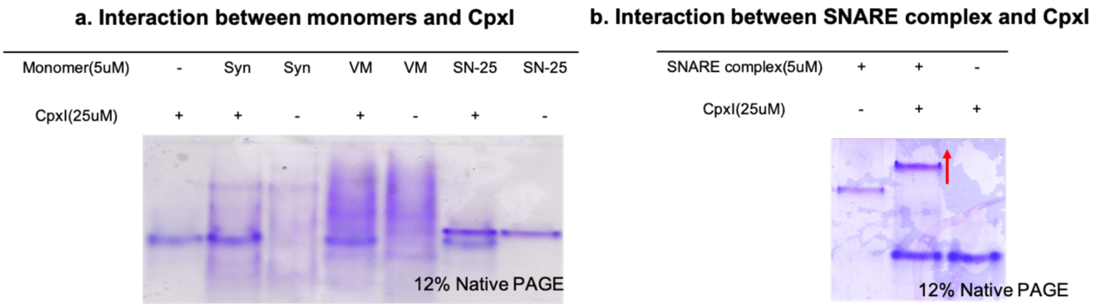

Supplementary Figure 3. Interaction test between SNAREs and CpxI. **a** Binding interaction between SNARE monomers and complexin-1. **b** Binding interaction between SNARE and CpxI on native page. CpxI could bind with SNARE complex.

5. Primers for the preparation of DNA handles

Primer sequences for the biotin- and digoxigenin-modified 2260-bp handles were:

SHP2 Primer: /5ThioMC6-D/ AAA TCG ACG CTC AAG TCA GAG GTG

Dual-DIG Primer: /5'Doubler Amino + DIG/ ATC ATC CAA GGC TGA GCC TGC AGG

6. Supplementary movie

Supplementary movie demonstrates how the single molecule DNA forms the tether between two microspheres. The DNA handle was attached to the left microsphere (red circle) through digoxigenin /anti-digoxigenin. The second microsphere coated with streptavidin was able to move towards the left microsphere through the movable trap. Once a tiny microfluidic flow was applied to the main channel from left to right, the DNA handle will float at the downstream, and the other end of the DNA with biotin molecule will attach to the downstream microsphere on the surface through streptavidin molecule. Once the tether was formed, the force will increase in presence of increase in trap separation, and the force will keep constant when the trap separation stops to increase.

7. Middle cross-linked constructs of SNARE complex

The half-zipped state of the N-terminal cross-linked SNARE complex is too short, so we needed to build a new SNARE pulling system with sable and long half-zipper state. To got four

different middle-cross-linked SNARE complex, we designed and purified four different mutants of syntaxin, and four different mutants of VAMP. The point mutations of syntaxin and vamp were chosen by pre-design on the base of X-ray structure of SNARE complex. The cysteine of wild-type syntaxin and vamp is mutant into alanine, and new cysteine is mutant at new sites. And we incubate the SNARE complex on 34 °C, over 8 hours with shaking of 300rpm to test the formation of intramolecular disulfide bond between syntaxin and VAMP. The red arrow at 30 kDa show the supported band, which confirm the existence of the middle-cross-link. Then we optimized the salt ion concentration, PH, temperature, oscillation speed and duration for the formation of disulphide bond, and finally selected the buffer of 150mM NaCl, PH8.5, and incubated at 300 rpm at 34°C for more than 8 hours. The connection efficiency as shown in Figure 3b is obtained (since the molecular weight of the complex is twice that of the syntaxin-VAMP, the connection efficiency of the -6 layer cross-linked SNARE complex is about 50%).

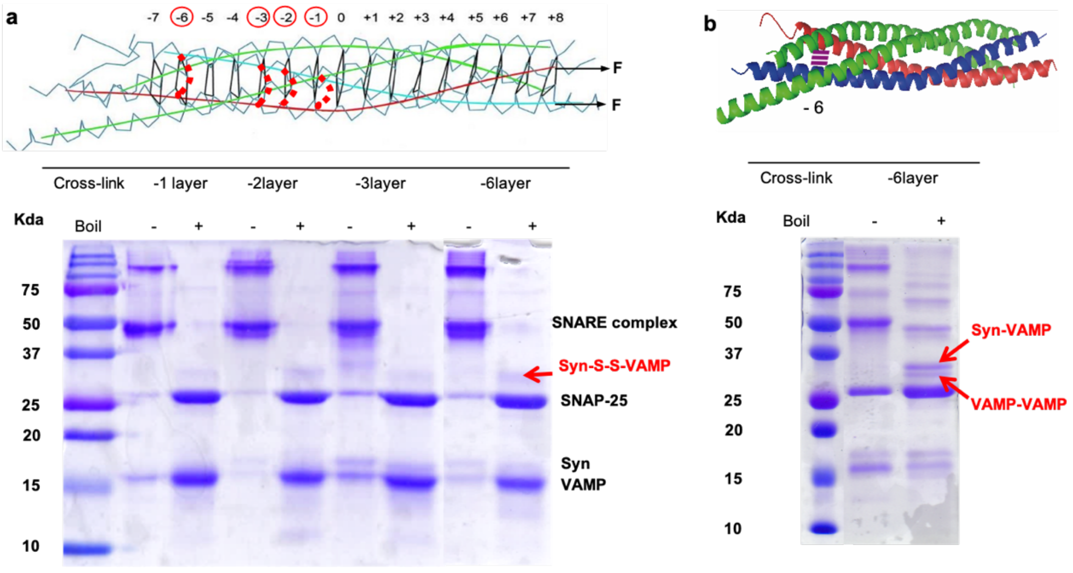

Supplementary Figure 4. Boil assay of intramolecular disulfide bond on SDS page. **a** -1, -2, -3, -6 on the top symbolizes corresponding layer of SNARE complexes. Red dashed lines repensent the disulfide bond between cysteines. **b** The efficiency of disulfide bond generation between Syntaxin and VAMP of -6layer SNARE complex was optimized.

8. Mutated protein sequence

8. 1 VAMP2 (1-92, Q36C)

SAGGMSATAATVPPAAPAGEGGPPAPPPNLTSNRRLQQTCAQVDEVVDIMRVNV DKVLERDQKLSELDDRADALQAGASQFETSAAKLKRKYWWKNGGSGNGSGGL CTPSRGGDYKDDDDK

8. 2 Syntaxin (172-265, L205C)

SAGGNPAIFASGIIMDSSISKQALSEIETRHSEIIKCENSIRELHDMFMDMAMLVES QGEMIDRIEYNVEHAVDYVERAVSDTKKAVKYQSKARRKKGGSGNGGSGSGLN DIFEAQKIEWHEDYKDDDDK

8. 3 SNAP25 (1-206, C85S/C88S/C80S/C92S)

MAEDADMRNELEEMQRRADQLADESLESTRRMLQLVEESKDAGIRTLVMLDE QGEQLERIEEGMDQINKDMKEAEKNLTDLGKFSGLSVSPSNKLKSSDAYKKAW GNNQDGVVASQPARVVDEREQMAISGGFIRRVTNDARENEMDENLEQVSGIIGN LRHMALDMGNEIDTQNRQIDRIMEKADSNKTRIDEANQRATKMLGSG

The sequences with underline were the native sequences, red marked amino acids were mutated amino acids, the FLAG tag (DYKDDDDK) at the C-terminal was used for protein purification, but was not used in this assay. SAGG at N-terminal and GGSGNGSGG at C-terminal were protein linkers, which were designed to increase the flexibility of protein.

9. Single molecule experiment on -2 layer and -6 layer cross-linked SNARE complex

We have tried all the four crosslinking sites (-1, -2, -3, -6), and found that the crosslinking at -1 or -3 layer was unstable, as a result, the efficiency to form a tether is very low, and the tether would easily break under a force of 16 pN. We have successfully formed tether with high efficiency for the crosslinking at -3 and -6 layers. Although the success rate is low for the crosslinked SNARE at -3 layer, we have successfully collected the dynamic transition signal at ~ 16 pN. In figure S5a, the de-assembly signals obtained by the experiments of the SNARE

complex of -2 layer was hopping at 10 nm, corresponding to the de-assembly process of C-terminal and partial N-terminal of 0 to -2 layer (about 28+8 amino acids in total). In figure S5b, at the same force, the de-assembly signal hops at 20nm, which correspond to the de-assembly process of the C-terminal of the SNARE complex and partial N-terminal of the 0-6 layer (about 28+22 amino acids in total). Because the -2 layer is too close to the 0 layer, and the de-assembly protein fragment covering the N-terminal is too short, insufficient for us to study the interplay of the Cplx with the N-segment of SNARE complexes. Therefore, the subsequent experiments are mainly based on the structure of the -6 layer with longer hopping.

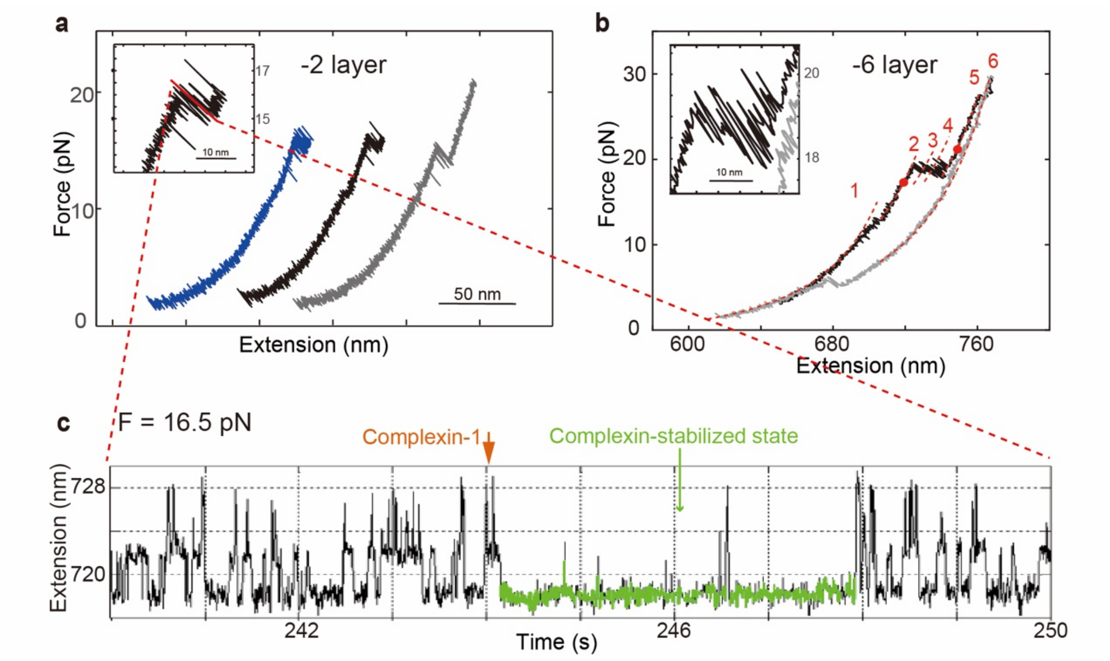

Supplementary Figure 5. Single molecule experiment on -2 layer and -6layer cross-linked SNARE complex. a. FEC of -2 layer cross-linked SNARE complex without Cpx. b. FEC of -6 layer cross-linked SNARE complex without Cpx. c. Extension-time trace of -2 layer cross-linked SNARE complex with Cpx.

We also recorded the dynamic transition of the -2 layer crosslinked SNARE complex. The -2 layer SNARE complex is stretched to a hopping signal in presence of a fixed trap separation. We observed the transition among three state of SNARE complex in equilibrium (Fig. S5c). Then CpxI was added through protein channels, and significantly long stay pauses were observed during the rapid transition from SNARE complexes (in green, Fig. S5c), where the

SNARE complexes were stabilized in a quad helical bundle state, revealing a new CpxI dependent state. This is consistent with the signal that the -6 layer SNARE complex could be stabilized in the four-helical bundle state (i.e., the C-terminal stabilized state in Fig. 2).

In summary, we have tried to crosslink the SNARE complex at various layers and verified that the -6 layer allows stable performance and maximum interaction region on the SNARE complex with the Cplx molecule. Although different crosslinking site shows slightly different force-extension graph (mainly the open distance is different), the interaction of the Cplx and SNARE complex in the same region suggests consistent mechanism. On the data interpretation, the major principle is the same, the only difference is that when we fit the force-extension curve with the worm-like chain (WLC) model, a different contour length for each of the four-helix bundle, polypeptide was used according to the crosslinking site. In order to maximize the interaction region on the SNARE, most of our experiment was conducted with -6 layer crosslinking on SNARE complex.

10. Chamber fabrication

Standard cover-slides were cleaned with dishwashing liquid, then washed with clean water, drained, and ultrasonic cleaned in deionized water for 5 minutes. After rinsing in deionized water, the slides were ultrasonic cleaned in anhydrous ethanol for 5 minutes, and then dried. The glass slide was cut with 6 inlets/outlets using the Laser engraving machine. Meanwhile, the parafilm was cut into three channels as described in Supplementary figure S2.

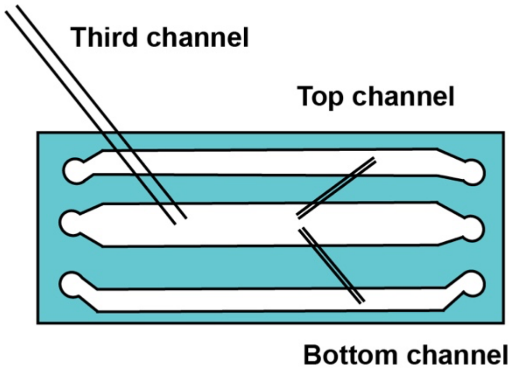

Supplementary Figure 6. Sketch map of chamber cell. It is constructed by sandwiching one p arafilm between two slides, with parafilm thickness as the sample storage space.

The custom chamber was made using the following steps：

(1) Place one parafilm on the slide without laser engraving, then mount the glass pipettes to form the connection channels between the top/bottom channels with the main channel. Put another pipette to serve as the protein channel if necessary. Then put on top of them another parafilm and coverslide, and gently press on the assembly. Such that the parafilms and the capillary are sandwiched in between.

(2) The sample pool is turned over and placed on the metal hot plate to melt the parafilm, and the appropriate pressure is applied to make the slide and the melted parafilm tightly bonded for about 20 minutes, and then it can be cooled after taking out.

(3) Assemble the chamber cell according to the diagram and install it on the optical tweezers instrument. Fill the chamber cell with 0.2% NaN_3 to prevent bacterial growth when finish the experiment.

When assembling the chamber, be careful to test the leakage using clean water, and make sure there will be no air bubbles inside the channels. The tiny air bubbles can be removed by gently tapping on the plastic tubing to generate gentle vibration.

11. Configurations of the SNARE with and without Cpx

In the absence of Cpx, The SNARE complex dynamically transits among 5 possible states depending on the magnitude of applied force. They are: State 1, SNARE complex fully folded; State 2, the linker domain open state; State 3, the C-terminal domain (CTD) open state; State 4, Middle Domain (MD) open state; State 5, N-terminal domain (NTD) open state. Those configurations are explicitly illustrated in the following figure.

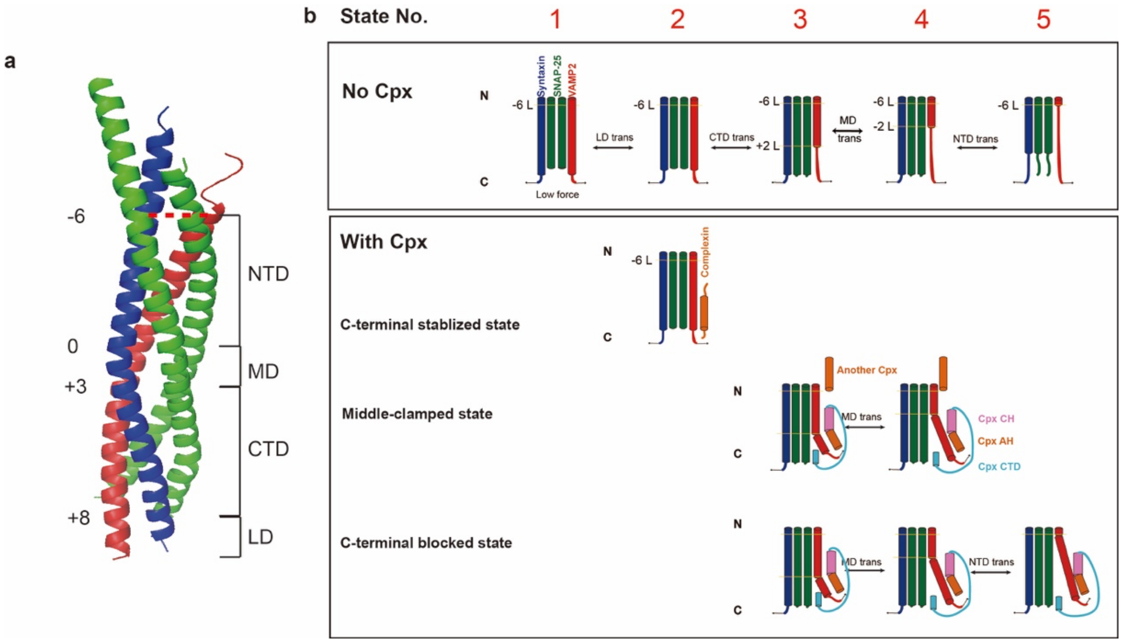

Supplementary Figure 7. Complete configuration of the SNARE complex with and without Cpx. a. Crystal structure and domain location in the SNARE complex. b. Illustration of the configurations of SNARE complex in the absence (top) and presence (bottom) of Cpx.

In the presence of Cpx molecule, due to the interplay of Cpx and the SNARE complex, the Cplx molecule may bind to different domains on the SNARE complex, and thus displays different configurations of the Cpx-SNARE complex. Briefly, we interpret our single molecule data into three groups.

(1) C-terminal-stabilized state: The CpxI molecule preferentially bound on the C-terminal of the VAMP2 component in the SNARE complex. Therefore, the at a force range of 15 ~ 19 pN, the original SNARE complex keeps at State 2 with merely the linker domain open. The SNARE complex cannot unfold the linker domain (State 3), middle domain (State 4), and N-terminal domain (State 5). This has also been confirmed by our single molecule experiment that only State 2 appears in the single molecule traces in the presence of CpxI.

(2) Middle-clamped state: When the CTD in SNARE opens, the CpxI molecule may clamp the SNARE NTD and CTD domains with two domains in CpxI. As a result, the SNARE complex keeps the linker domain (LD) open, and can only hop between the middle domain (MD) folded and unfolded states.

(3) C-terminal blocked state: While the Cpx molecule only clamp the CTD of SNARE complex, the SNARE complex may hop among linker domain (LD) open state, middle-domain (MD) open state, and C-terminal (CTD) open state.

In summary, based on the explanation, the so-called “C-terminal stabilized”, “Middle-clamped”, and “C-terminal blocked” states demonstrate the function of Cpx molecule on the SNARE complex, while State 1 through 5 denote four different configurations of SNARE complex. They are associated but not exactly equal.

12. Definition of signal rate

The fragments of or complete CpxI interacts with the SNARE complex, and the Extension trace of the SNARE complex may display three different states, i.e., the C-terminal stabilized state (CT), middle clamped state (MC), and the C-terminal Blocked state (CB). Here the probability to each state is defined as the signal rate,

$$\gamma_i = \frac{N_i}{N_{CT}+N_{MC}+N_{CB}} \quad (9)$$

Here, $i = CT, MC, CB$, and CT, MC, CB, represent C-Terminal stabilized, Middle-Clamped, C-terminal Blocked states, respectively. This definition has been used to process the signal rate in Fig. 4d, and Fig. 5c in the main text. Especially, there was only C-terminal inhibited state for CpxI 48-73aa in Fig 5a.

For we injected only buffer (first column in fig.6a) though protein channel as control, and the majority (81% of 31 molecules under test) showed no change after the injection. And once the fragment 83-134 aa (mostly part of CTD, without CH) was injected, and 18 of 27 SNARE complexes showed no change (second column of Fig. 6a), which was a negative control of experiments in Fig. 5. In Fig 6a, we define the signal rate as,

$$\gamma_i = \frac{N_i}{N_{Ct}+N_{MC}+N_{CB}+N_{NC}} \quad (10)$$

$i = CT, MC, CB, NC$. Here, CT, MC, CB, NC, represent C-Terminal stabilized, Middle-Clamped, C-terminal Blocked states and No Change molecules, respectively.

13. HMM analysis of C-terminal stabilized state and middle clamp state

In the presence of 8 μM full-length CpxI, 49 SNARE complexes changed their state after the addition of CpxI. We observed that SNARE complex could be clamped by CpxI into the exactly half-zippered state (middle-clamped state, 7 molecules, 14% signal rate, Fig. 2a, 2b, 2d). Accordingly, the dynamic folding of SNARE complex only took place between 3~4 states at this situation. Surprisingly, the SNARE complex can also maintain in C-terminal stabilized state after the addition of CpxI (C-terminal stabilized state, 20 molecules, 41% signal rate, Fig. 2a, 2b, 2d). Accordingly, SNARE complexes have changed from hopping among 2-5 states to being maintained only in the second state with the addition of CpxI.

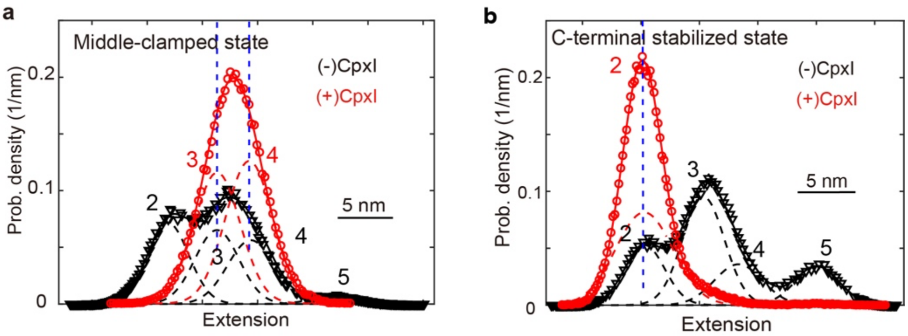

Supplementary Figure 8. HMM analysis of C-terminal stabilized state and middle clamp state. Probability distributions of the extensions correspond to the traces in Fig 2a (a. middle-clamped state; b. C-terminal stabilized state) in the presence (red circle) and the absence (black triangle) of CpxI and their best fits by a sum of four Gaussian functions (red and black lines).

14. Unfolding force statistics of SNARE complex with full-length and 1-83aa Cpx

We also counted the unfolding force of the SNARE complex in the absence and presence of either full length CpxI or the fragment 1-83 aa. The hopping takes place at ~21 pN in the absence of CpxI, while hopping happens at a higher force level in presence of full or fragmental CpxI. The maximum likely force increased from ~21 pN to ~24 pN and some events happen at even higher force of ~43 pN in the presence of full length CpxI (top panel in Fig. S7).

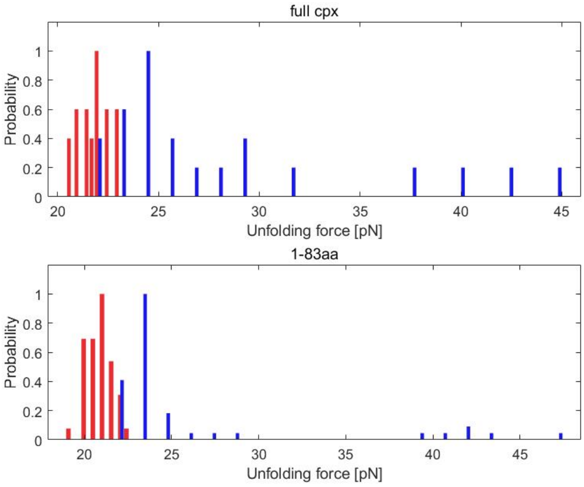

Supplementary Figure 9. Unfolding force statistics of SNARE complex with full-length a nd 1-83 aa Cpx. The red bar shows the unfolding force histogram in the absence of the CpxI, while the blue bar suggests the unfolding force histogram in the presence of CpxI.

Although similar phenomenon has been observed with the fragment 1-83 aa of CpxI (bottom panel in Fig. S7), it is worth to note that there are more high-force signals in the full-length Cpx, which may imply more stable binding of full-length Cpx to SNARE complexes.

15. The stabilization function of CpxI NTD

To pinpoint the possible role of the NTD in the CpxI -dependent SNARE disassembly, we removed the NTD in the CpxI construct. In the presence of CpxI 26-83 aa, 38 dynamic molecules changed their state after the addition of CpxI (Fig. 4a, 4b). Interestingly, the extension-time traces of 25 molecules (66%) were still stabilized at C-terminal stabilized state (Fig. S8). The rate of C-terminal stabilized state decreased dramatically, showing that the removal of NTD caused significant changes in the CpxI-dependent SNARE disassembly (Fig. 4d), which indicates that the CpxI stabilizes the SNARE complex critically depending on its N-terminal domain (NTD, 1-26 aa).

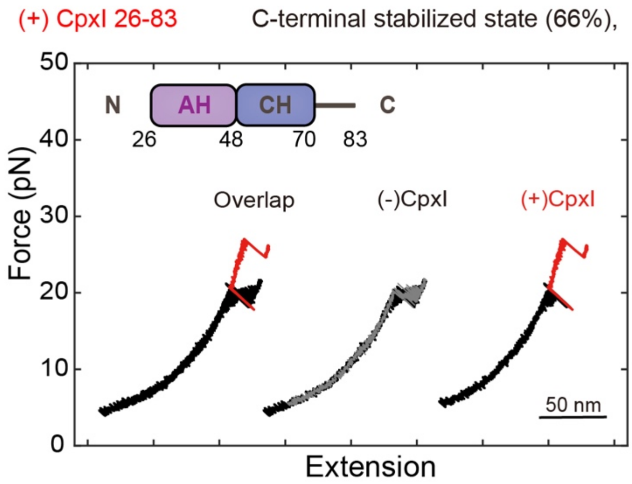

Supplementary Figure 10. The stabilize function of CpxI NTD. In the presence of 8 μM 26-83 aa, 57.5% of 66 transition-state molecule changed their state after the addition of Cpx. FECs of single SNARE complexes under constant forces showing that 66% of them was stabilized to linker-open state after (red) the addition of 8 μM 26-83aa in real time.

16. No interaction between 1-83 aa and 83-134 aa

In order to test whether mixed CpxI 1-83 aa and 83-134 aa would interact in advance, we conducted Ni-NTA pull-down experiment. The 1-83 aa without his-Tag was mixed with sumo_83-134 aa with 6* His Tag for half an hour, and then incubated with Ni-NTA for 1 hour. Collect the post-cleaning solution of the flow through fluid and the low imidazole buffer, and finally elute with the high imidazole buffer. Obviously, almost all 1-83 aa flowed out before elution (Supplementary Figure 9, lane 5 and 6, FT: Flow Through), while there was no 1-83 aa in the hyperimidazole eluent, only SUMO_83-134 aa (Supplementary Figure 9, lane 7), proving that there was no interaction between 1-83 aa and 83-134 aa.

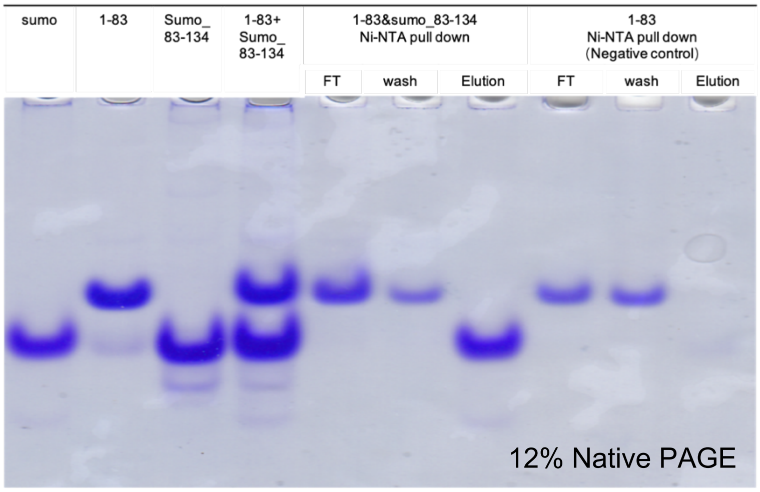

Supplementary Figure 11. Ni-NTA pull-down assay of interaction between 1-83 aa and 83-134 aa. Mixture of sumo_83-134 aa (with 6*his-tag) and 1-83 aa was incubated with Ni-NTA for 1 hour. Then we collected the flow-through and wash solution, finally eluted the beads by buffer with 250 mM imidazole. Obviously, almost all 1-83 aa flowed out before elution, indicated that there was no interaction between 1-83 aa and 83-134 aa.

17. CpxI CTD can inhibit the assemble of C-terminal of SNARE complex and stabilize the N-terminal of SNARE complex.

We introduce CTD fragment (83-134 aa) combined with 8 μM 1-83 aa of CpxI to interact with the SNARE complex during dynamic assembly. This scheme is similar to the addition of full length CpxI, but each CpxI molecule is separated into two pieces. Unexpectedly, 18 SNARE complex molecules changed their states after the addition of 8μM 1-83 aa fragment and 8μM 83-134 aa (with molar ratio of 1:1); 21 SNARE complex molecules changed their state after the addition of 8 μM 1-83 aa and 16 μM 83-134 aa (molar ratio 1:2); 22 SNARE complex molecules changed their state after the addition of 8μM 1-83 aa and 24μM 83-134 aa (molar ratio 1:3). Figure S10 show the FECs of C-terminal stabilized state (Fig.S10a), C-terminal blocked state (Fig.S10b), middle clamped state (Fig.S10c) after the addition of CpxI.

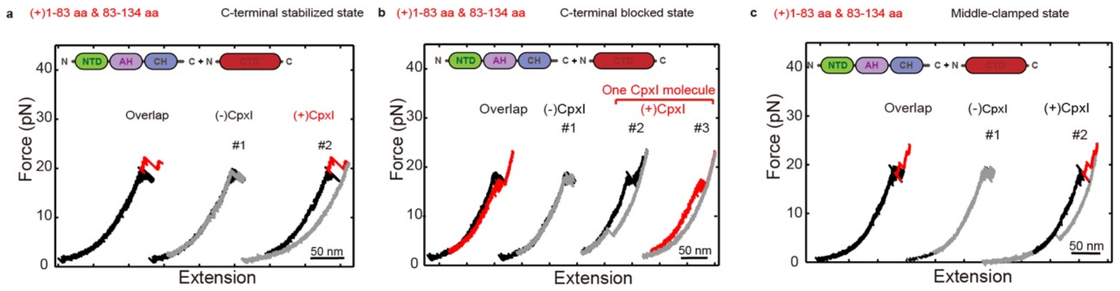

Supplementary Figure 12. CpxI CTD can inhibit the assemble of C-terminal of SNARE complex and stabilize the N-terminal of SNARE complex. **a-c** FECs of single SNARE complexes under constant forces showing SNARE unfolding kinetics before (black) and after (red) the addition of 8 μM 1-83aa and 8/16/24 μM 83-134aa in real time.

18. Recipe for Oxygen scavenging system

Name	Stock concentration	Stock buffer	Temperature (°C)	Working concentration
Glucose	400 mg/ml	Water	-20	250 mg/ml
Glucose Oxidase	50 mg/ml	50 mM NaAc (pH 5.1)	-20	37 mg/ml
Catalase	20 mg/ml	50 mM KPB (pH 7.0)	4	1.7 mg/ml

Supplementary Table 2. Experiments were carried out at room temperature (22 °C) in the HEPES buffer (25 mM of HEPES, 150 mM NaCl, 0.02% CA630, pH 7.4), supplemented with 2% oxygen scavenging system (for reduce light damage to hold for longer time).

Supplementary References

1. Chen, X. C.; Tomchick, D. R.; Kovrigin, E.; Arac, D.; Machius, M.; Sudhof, T. C.; Rizo, J., Three-dimensional structure of the complexin/SNARE complex. *Neuron* **2002,** *33* (3), 397-409.
2. Bracher, A.; Kadlec, J.; Betz, H.; Weissenhorn, W., X-ray structure of a neuronal complexin-SNARE complex from squid. *J Biol Chem* **2002,** *277* (29), 26517-23.
