# Supplementary material for: Complexin-1 regulated assembly of single neuronal SNARE complex revealed by single-molecule optical tweezers
Source: Commun Biol. 2023 Feb 7;6:155. doi: 10.1038/s42003-023-04506-w (PMC9905088; doi:10.1038/s42003-023-04506-w)
Supplement: Supplementary file 3 — Description of Additional Supplementary Files [file 42003_2023_4506_MOESM3_ESM.pdf]

Description of Additional Supplementary Files

File name: Supplementary Movie

Description: Supplementary movie demonstrates how the single molecule DNA forms the tether between two microspheres.
